# An Error in Medical Writers

**Published:** 1872-01

**Authors:** L. B. Bouchelle


### AN ERROR IN MEDICAL WRITERS.

A brief note of *attention*, brethren of the profession, rather than of *criticism*. After reading the books and the periodical literature of the profession for sixteen years, we hope to be allowed to call the attention of medical writers to what is a manifest error, viz : the use of indefinite terms, especially in therapeutics. Most persons read the journals with a view of being benefitted by the wisdom, experience and skill of others, and it frequently happens that a good remedy is rendered worthless to the reader by the use of such indefinite terms as "same," "a little," "a small quantity," a "few grains," and very commonly, "small doses." These terms all have such a latitude that no one can arrive at what is meant. Medicine, like music, has an universal language, and it is simple, easy to be learned and remembered, and why not use it? There is another class of errorist, among medical writers, whose "pure minds" should be stirred up at this point, viz: Those who are afflicted with pedantry. Such writers always fill up their articles with the most strained technicalities. Now (though ostensibly writing in English), they quote French, now Greek, then Latin and German, and that too, in dealing with the nomenclature of diseases, remedies, and worst of all, of chemistry and pharmacy.

It is true, some medical students have an opportunity of learning these various tongues, but the great mass of physicians are men of one tongue only, and this one is more than most men learn and use wisely. It has not been long since a very intelligent young physician brought to the writer a most excellent clinical work with a cramped expression, saying, "here is a book said to be excellent, been highly recommended to me, and all that, but owing to the frequent use of terms I do not understand, it is worthless to me, do take it and analyze these phrases and weights and measures." This is an instance of many. And this same man had had, and improved, fine educational opportunities, but he had not been to Paris or Berlin. Come, brethren of the quill, before submitting your articles to the editors and publisher, trim closely, and expunge everything smacking of indefiniteness or pedantry. Let us all use our own dialect, and not be speaking in proverbs or unknown tongues. Let us address ourselves rather to the task of simplifying rather than mystifying our articles, so that the reader may peruse to edification and not to *mistification*. Let us be to the point, and use language expressive of our *meaning* rather than our *learning*.

L. B. BOUCHELLE, M. D.

